# School–Family Relations: An Educational Challenge in Times of COVID-19

**DOI:** 10.3390/ijerph182010681

**Published:** 2021-10-12

**Authors:** Mario Ferreras-Listán, Coral I. Hunt-Gómez, Pilar Moreno-Crespo, Olga Moreno-Fernández

**Affiliations:** 1 Department of Experimental and Social Sciences, Faculty of Educational Sciences, University of Seville, 41013 Seville, Spain; mferreras@us.es; 2 Department of Language Teaching, Faculty of Educational Sciences, University of Seville, 41013 Seville, Spain; coralhuntg@us.es; 3 Department of Research Methods and Education Diagnosis, Faculty of Educational Sciences, University of Seville, 41013 Seville, Spain; pmcrespo@us.es

**Keywords:** COVID-19, emergency education, families, information and communication, educational challenges

## Abstract

The COVID-19 pandemic has widened the gap regarding access to educational opportunities, which was included in the Millennium Development Goals (MDGs). This descriptive, quantitative study aims to examine the communication strategies employed by secondary schools in Spain during the lockdown, as well as to analyse the co-responsibility of the educational process between schools and families. An ad hoc questionnaire (GIESBAFCOV-19) was designed and implemented to gather information. The results show that, in most cases, mothers were responsible for assisting and supervising their children’s homework as persons in charge of education-related matters. Additionally, before the lockdown was put in place, about half of the participating families received information from the educative centres regarding the disease and sanitary measures. Once the lockdown took place, families put the focus on their children’s schoolwork, not without difficulties in academic and digital literacy. In general, the families were satisfied with the communication established with the educational centres. The present study has raised the necessity to improve communication between centres and families and to reflect on the tools and systems used for its exchange. Consequently, it seems that information and digital competences should be promoted to guarantee an equalitarian education for all.

## 1. Introduction

During the last decade, the United Nations (UN) promoted the Millennium Development Goals (MDGs), which included challenges addressing climate change, economic inequality, innovation, sustainable consumption, peace and justice, and access to education. This institution is currently working on the Sustainable Development Goals (SDG). SDG 4, Quality Education, is focused on ensuring inclusive, equitable and quality education and promoting lifelong learning opportunities for all. This call for action to all countries to develop in global partnership is a challenge of great social impact with a clear humanistic and moral perspective.

However, the world was not prepared for the pandemic situation we were entering when a new coronavirus outbreak emerged in late 2019 in Wuhan (China). The disease caused and continues to cause many deaths, along with a rapid global spread that activated anti-COVID protocols [1]. Among these anti-COVID guidelines were those issued by the Spanish Ministry of Health on 12 February 2020, which called for the implementation of general infection control measures, recommending frequent hand washing and correct respiratory hygiene [2]. The repercussions at the educational level initially materialised on the 8 March 2020 with the publication of the Guide for schools and educational centres in the event of COVID-19 Cases [3], which established specific guidelines for preventing COVID-19 infection and action guidelines in the event of suspected or confirmed cases of infected students. Among the general principles were the following: the use of facemasks in educational settings was not recommended; sick people showing symptoms of infection were urged not to attend school and to avoid contact with healthy people; extra hygiene measures were requested and increased frequency of handwashing, especially after coughing, sneezing, touching or handling tissues; and special attention was requested when leaving and arriving home, at school, after using the toilet, during sports activities, and when preparing or eating food. It was also indicated to avoid, as far as possible, touching your eyes, nose, and mouth, and to cover the mouth with a handkerchief when coughing and sneezing, or, if that is not possible, coughing and sneezing into the inside of the elbow. One of the noteworthy aspects of the Guide for schools and educational centres in the face of cases of COVID-19 [3] is that it includes new tasks for teachers and educational support staff, such as familiarising the educational community with the main precautionary measures and the dissemination of prevention strategies and health and hygiene. Therefore, schools should inform students and their families about these crisis-linked aspects.

As the extraordinary health persisted, following the detection of significant community transmission of the virus, on the 12 March, the measures already established in some areas were extended to the rest of the country, which resulted in suspending classroom teaching at all educational levels as well as complimentary educational activities [4]. This affected other sectors apart from education, since on the 14 March, a State of Alarm was declared in Spain, and, together with it, the Spanish government established the lockdown of the entire Spanish population in their homes, only allowing leaving their houses for those activities categorised as essential [5]. The new health context entailed challenges at different levels and in different areas, and this greatly affected the field of education [6]. Firstly, education moved from the classroom to the family home and from face-to-face classes to virtual learning [7,8]. In this crisis context, correctly managing communication was essential. Therefore, schools had to adopt emergency solutions to provide telematic responses to both students and their families.

These exceptional circumstances, together with the improvised planning to meet the new socio-educational needs, resulted in numerous requests for information on the health alert situation and its management addressed to schools. In this regard, a guidance counsellor at a Secondary school described how her school received a high number of e-mails from families, and they had to reconsider priorities to be prioritised during the lockdown. It must be borne in mind that education professionals faced numerous labour difficulties during the lockdown, which were added to the need of managing new pandemic-related issues as well as the optimal brokering of information into the families [9].

Inevitably, even if it was considered necessary to stop the spread of the virus, educational institutions’ shutdown had repercussions at all levels. Among them, difficulties in accessing learning opportunities were increased, as not all families had the necessary resources to comply with the teaching–learning processes at home [10]. This sudden school and social termination affecting Secondary Education students, considering the particularities of their age, may have led to psychological consequences caused by social isolation [11]. Considering the situation at that time, despite its drawbacks, the closing of face-to-face educational institutions was deemed as a vital measure to avoid the spread of the virus [12].

Regarding the implementation of the SDG 4th Goal, the pandemic context exposed gaps in the education systems to promote inclusive, equitable and quality education as well as to provide equal access to learning opportunities. Therefore, it is important to continue working for the Millennium Development Goals. In this regard, the pandemic and the limitations to only communicate with the families virtually generated a unique situation that compelled schools and their staff to develop ad hoc information management solutions. The lack of recommendations regarding communication management or the information that needed to be delivered to the families during lockdown did not improve the situation. All this led to a first adaptation phase that took place during the first lockdown weeks, in which educational centres had to communicate all the necessary information to the families to continue the teaching–learning process at home.

### 1.1. Crisis Communication in the Field of Secondary Education

Crisis communication is a field of study that has become vitally important during the COVID-19 social and health emergency. Adequate transmission of information is paramount in times of crisis, as it is conveyed during extremely difficult situations where the population needs to cope with the perception of danger and negative feelings [13]. Although each crisis has its own characteristics, all crises share some common elements that affect the reputation of the organisation in charge of providing information [14]. For a good communication strategy, it is necessary to follow some simple rules for information management in crises, i.e., communicating with credibility and confidence, taking responsibility for the information provided, having a spokesperson, avoiding lies, inexact information, silence and improvisation [15].

In order to provide solutions as quickly as possible during the lockdown, the Spanish Ministry of Education and Vocational Training, in association with the different heads of the seventeen Autonomous Communities, developed some specific strategies. Thus, communication channels were established among all the educational agents: the administration, the regional education boards, primary and secondary schools and universities. However, since they were conveyed to the staff some weeks from the beginning of the lockdown, and some of the staff had already started the communication with the pupils and their families, these strategies regarding information management were not fully complied with.

Due to the democratisation of the Internet and the spread of the use of social networks and instant messaging, the information during the COVID-19 was received very differently. Never had humanity experience a crisis of a similar magnitude in which people were more informed. However, with the proliferation of fake news on social networks [16], the need for truthful and reliable information increased proportionally. People’s information consumption exponentially increased during the lockdown, as did criticisms towards the media [17]. Thus, citizens used the Internet to cover their needs regarding health information [18]. It can be stated that crises lead citizens to actively seek information according to their interests through different channels; however, when dealing with educational needs, they use more traditional ones [19], which renders information management carried out by education institutions particularly interesting.

Humanity faced an enormous challenge as we were facing an unprecedented crisis, and educative centres had no previous experience when dealing with these situations. Nonetheless, it was clear that the main priority was ensuring that no one was left behind, that there was no student whose family had not been contacted by their centre. During the uncertainty experienced first weeks of the pandemic, it was essential to establish communication mechanisms between the centre and the families to avoid interrupting the learning process and ensuring that students continue their education at home [20].

Before the pandemic, families and centres shared responsibility was considered important [21] for guaranteeing the Right to Education; the COVID-19 pandemic experience during 2020 proved it to be essential.

### 1.2. The Role of Families in Education during School Closures

The sudden stop of face-to-face classes made families tend to their children’s educative needs at home and obliged them to get familiarized with many digital and technological mechanisms and devices. As Sosa-Díaz [22] highlights, poor parental computer skills and low academic and digital literacy at home can aggravate educational inequalities among students. Therefore, during the lockdown, academic outcomes became more dependent on motivation, attention, attitude, parental skills and accompanying strategies. Undoubtedly, the gap in access to education became obvious since some families did not have the appropriate technological means [23,24] or could not assist their children due to a lack of availability or capacity [25]. Associated economic, health and other problems need to be considered as they have a clear repercussion on how education is managed. In our knowledge, society, access to learning, or the lack of it can make all the difference to a person’s future [12].

From the perspective of families and educators, school closure raised many questions about the students’ socio-educational development and the need to implement several changes in the learning and assessment processes.

Although many studies have been carried out on education in pandemic times, specifically on issues related to teachers, students and virtual teaching [6,7,20], only some studies have focused on families. The studies [12,21] were crucial in the adaptation process during the lockdown and made many additional efforts to maintain communication during the COVID-19 crisis. The purpose of this paper is to study the communication strategies employed by secondary schools in Spain when they were closed due to COVID-19 from the perspective of the students’ families. It also analyses the issue of the schools and families’ shared responsibility during the educational process.

## 2. Materials and Methods

This work is part of a project on information management in Secondary Education about COVID-19 from the perspective of the students and their families. Specifically, it focuses on how Andalusian secondary schools have managed information from the perspective of the students’ families during the COVID-19 crisis, specifically during the lockdown in which schools were closed. For this purpose, a descriptive, quantitative methodological design was chosen.

### 2.1. Participants

A snowball convenience sampling technique was used. The questionnaire was sent to people in the research team’s environment whose children were in secondary education. Some of the respondents belonged to parents’ associations (AMPA). The questionnaire was shared through social networks and online communication systems, indicating that it should be disseminated to widen the sample.

The research sample consisted of 174 families, with teenage students in the educational stages of Secondary Education in the autonomous community of Andalusia. Some demographic aspects of the sample were noteworthy. Of the 174 responses obtained, 87.4% (*n* = 152) were implemented by mothers, while 12.6% (*n* = 22) were implemented by fathers. This trend coincides with those found in the other studies [26], which proved that education-related tasks are generally carried out by mothers. While 48.8% (*n* = 84) of the participating families stated that one or more members of the family were working at the time of lockdown, 22.7% (*n* = 39) of the families surveyed stated that one of the two parents had been affected by a Temporary Redundancy Proceedings (ERTE). The remaining (*n* = 49) 28.5% reported other family circumstances such as being unemployed prior to COVID-19, doing housework, teleworking, among others. As for the centres included in the study, 84% (*n* = 146) are public, 8% (*n* = 14) are subsidised centres, and the remaining 8% (*n* = 14) are privately owned.

### 2.2. Instrument

An ad hoc measurement instrument was designed and registered under the name of GIESBAFCOV-19 for gathering relevant information. This instrument consists of a questionnaire that analyses three dimensions: a first informative dimension related to the family circumstances in which they found themselves during the COVID-19 crisis; a second dimension related to the crisis communication management by Secondary Schools; and a third and final dimension analysing educational management.

The questionnaire has 22 items, of which 21 are multiple-choice questions and 1 is an open-ended question (Table 1).

The final version of the questionnaire experienced a double validation. In the first phase, it was validated by four experts in the field of education, who were researchers and professors from Spanish universities. Adjustments related to linguistic expression were made to facilitate the understanding of families and to guarantee the internal consistency of the instrument. In a second phase, a pilot test was carried out with four families with children in Secondary Education to ensure that the terminology was known and understandable.

The information was gathered during April 2020, during the COVID-19 crisis and two weeks after the start of lockdown in Spain. We use the *Google Forms* application (https://forms.gle/kStXD7paQWBPmPi29) (accessed on 1 October 2021) and disseminated via email and instant messaging in various educational centres in Andalusia. Participation was completely voluntary, and participants’ data were anonymous. The estimated time to answer the questionnaire was 15 min, and once the survey was completed, the respondent was given the opportunity to share it with other people via instant messaging, creating a snowball effect. Data were extracted from the collected responses and subsequently coded in the SPSS data editor version 25 for further analysis.

## 3. Results

### 3.1. Crisis Communication Management (GCOMC)

As to whether the school implemented measures to prevent the spread of COVID-19 prior to the closing of schools, as indicated in the *Guide for schools and educational centres in cases of COVID-19* published by the Health Alerts and Emergencies Coordination Centre, 42.5% (*n* = 74) of the participating families stated that basic hygiene measures, such as washing hands thoroughly or coughing within the elbow, were taught in secondary schools. 27.6% (*n* = 48) of the families stated they were not sure, while 28.2% (*n* = 49) indicated that the school was not working on measures to prevent the spread of COVID-19 they were closed. 1.7% (*n* = 3) of the participants did not answer. Therefore, and despite the official guidelines, more than half of the participating families indicated that they could not affirm that the centres were complying with the instructional recommendations regarding the prevention of COVID-19 infection.

Regarding the way schools communicated with their student’s families (Table 2), the results obtained show that 89% (*n* = 155) of the families stated that they had been informed by the schools, either occasionally (43.1%, *n* = 75)) or many times (45.9%, *n* = 80). Likewise, 1.2% (*n* = 2) of the families specified that they had been informed, although not directly by the school, but by the AMPA or by the parent class delegate. Therefore, more than 90% of families received information about the health crisis from the school or through a member of the educational community. However, it is noteworthy that 9.2% (*n* = 16) of the families stated that they had not been informed.

This study also aims to examine the ways and means by which information is conveyed to families in crisis situations, as well as the number of persons who do so their position in the centre. As official guidelines were established once the lockdown had already taken place, it is of particular interest to analyse the way in which staff organised to inform the families and the students as it will be most useful for drawing conclusions and action guidelines in case they are needed in the future.

In order to establish a profile of the staff and members of the educative community who contacted the families to inform them about issues related to COVID-19, two aspects were considered: the number of people who established communication from the centre with families and their position.

Thus, information was conveyed during lockdown as follows: 43.7% (*n* = 76) indicated that only one person had contacted them; 23.6% (*n* = 41) stated that they were contacted by two centre-related people; 15.5% (*n* = 27) by at least three people; 6.3% (*n* = 11) by at least four; and 1.7% (*n* = 3) indicated that up to five different people related to the centre contacted them to inform them about the COVID-19 crisis. The results show that a high percentage of families (around 47%) received information from the centre through several people, in some cases up to five. Previous research advocates for the need to unify interventions in the figure of a spokesperson, as a multitude of voices to transmit information generates confusion and a lack of trust. This situation can be due to the suddenness of the COVID-19 crisis, the lack of official indications and guidelines and a lack of internal coordination, which may have been perceived by the families as stressful, as they were over-informed, which the WHO came to describe as *infodemic*. It should also be noted that 15 families (8.6%) did not answer this question, which may mean that they were not clear whether the centre contacted them directly.

Data revealed that in 41.9% (*n* = 73) of the cases, it was a member of the school management, together with other teachers and/or representatives of the families, who had contacted the families. In 33.9% (*n* = 59) of the cases, it was exclusively a member of the school management. In 8% (*n* = 14), it was the student’s tutor, while in 7.5% (*n* = 13), it was someone linked to the families, either a member of the AMPA or a parent class representative. Fifteen families again did not answer this question. In one of the cases, it is indicated that the school psychologist contacted the family.

In the light of these data, it is established that the options taken to communicate with families from the educational centres are heterogeneous, although in most cases (around 75%), it was the management staff who were centralising this task, either individually or with the collaboration of other agents in the educational community. Moreover, it appears that they do not follow any pre-determined instructions or procedures.

Another aspect that still seems to be undefined and which also comprises the second dimension of the study is how the schools established communication with families (Table 3). In this sense, and as with the number of people who contacted families, the results highlight a certain lack of prior organisation and guidelines. Nearly a third of the participating families, 31% (*n* = 54), claimed to have received information through a single channel, which, in principle, seems easier to manage and involves less over-information [15]. However, in four cases, families were informed through five different channels, which probably led to the repetition of information and difficulties in managing it. In a similar situation, 34.5% (*n* = 60) of families were informed by three or more channels.

As for the telematic communication channels that were most used by the centres to contact families during the health crisis, they were offered several options of responses and the possibility of adding others. Thus, although the number of families surveyed was 174, a total of 368 responses were collected. Of all the responses obtained, two are particularly relevant. On the one hand, the PASEN module of the Seneca educational portal, which enables the communication between educational centres and families, legal guardians, and pupils in Andalusia, with 30.7% (*n* = 113) of the responses indicated. On the other hand, 23.1% (*n* = 85) of the families were contacted via the centre’s institutional e-mail. The remaining 43.3% (*n* = 159) received information through various media, including specific educational applications, instant messaging, and the school’s social networks.

The information received by the families is heterogeneous, ranging from health to psychological or academic information (Table 4). Once again, families were allowed to give multiple responses, bringing the total number of responses to 466. The results indicated that two issues were given particular importance: health-related and academic ones.

Regarding health-related issues, 20.4% (*n* = 95) of the responses from the families surveyed stated that the communications held addressed the health crisis and the social and hygienic distancing measures established getting infected and the spread of the virus. On the other hand, 7.3% (*n* = 34) reported having received specific information about the COVID-19 disease and its effects. In addition, 9.9% (*n* = 46) said they had received basic advice on how to act at home (washing hands, coughing into the elbow, etc.), and 5.1% (*n* = 24) stated being informed about strategies for keeping good mental health during the lockdown. Thus, around 42.7% of the communications made to families were linked to health issues.

The second topic that raised more interest was academic issues. In this sense, 29% (*n* = 135) of the families’ responses indicated that they received instructions on the academic tasks to be carried out by the students during the lockdown, as well as references to complementary educational platforms for the students’ training (16.5% *n* = 77). Only 0.2% (*n* = 1) of the sample stated having received information about administrative procedures.

### 3.2. Education Management (GESED)

In relation to the Education Management dimension, when examining the tasks set for students received online by their families during the lockdown, the results show that some subjects were given more attention than others. 97.1% (*n* = 167) of families reported having received homework in Mathematics, 95.9% (*n* = 165) reported homework in Language and Literature, 91.3% (*n* = 157) in Foreign Language, and 87.8% (*n* = 151) in Geography and History. These subjects, considered fundamental in Secondary Education, reached the highest values.

In 21.3% (*n* = 37) of cases, it was the educational centre that coordinated and supervised the homework through virtual sessions. In 34.5% (*n* = 60) of the cases, this task was completed exclusively by the mother, while in 14.4% (*n* = 25), mothers had the support of the school’s teaching staff. Exclusive fathers accounted for 2.3% (*n* = 4) and with teacher support for 4% (*n* = 7). In 21.8% of the cases, it was the students themselves who independently took responsibility for carrying out their homework. The results revealed that on two occasions, it was the older siblings who were responsible for supervising academic tasks.

Regarding which were the main obstacles encountered by the families in coping academically with the situation of lockdown, 27% (*n* = 47) of the cases indicated that they did not really encounter any obstacles that prevented the academic development of their children, while 73% (*n* = 172) did have difficulties and encountered various obstacles. Thus, 28.2% (*n* = 49) indicated that in some subjects they did not have the necessary knowledge to be able to help their children, while 19.5% (*n* = 34) indicated that the excessive number of activities entrusted to them prevented them from attending to their children adequately. They also indicated that the number of personal responsibilities (household chores, professional issues, etc.) stopped them from dedicating the necessary time to their children’s academic tasks. On the other hand, 6.3% (*n* = 11) identified the obstacles with the lack of necessary technological tools (computer, tablet, Internet connection, etc.) or that they felt apathetic and blocked when facing the COVID-19 situation (3.4%, *n* = 6). Finally, twelve families (6.9%) stated encountering various obstacles but that they gradually overcame them as they arose, without specifying what they were.

In relation to the perception of the information received regarding their children’s learning, as well as the assessment carried out by the teaching staff during the crisis and lockdown, the families indicated that in 45.6% (*n* = 78) of the cases, the teaching staff had adequately informed them about the teaching–learning process of their children through various channels (e-mails, video conferences, messaging applications, etc.). However, 54.4% (*n* = 93) of the families stated not having been duly informed during the school year about their children’s learning, limiting this information exclusively to the report card and the grades that were included in it. In this sense, the families complained about the process as deficient and arrived late, as they were not aware of whether they had to intervene to correct situations of poor school performance since they did not have the information.

As for the assessment carried out by the teaching staff, families were asked whether they considered it appropriate, due to lockdown and general crisis, to be more flexible and less demanding than in previous years. Of the participants, 44.8% (*n* = 77) of the families answered yes, that, due to the special circumstances and the added difficulties, the teaching staff should carry out a more flexible assessment, even lowering the level of demand in the subjects. In addition, 5.7% (*n* = 10) even stated that they all deserve a pass mark and that after the lockdown, they should return to normal educational practice. On the other hand, 31.6% (*n* = 55) of the families responded no, that the assessment and grading should remain the same but be adapted to the situation of not being in the classroom. Moreover, 11.5% (*n* = 20) indicated that they do not have an opinion on the matter, so they do not know whether the assessment should be the same or less rigorous. Finally, 5.7% (*n* = 10) indicated another option, stating, for example, that only the first and second trimester should be assessed, omitting the third trimester (where most of the lockdown took place). Another opinion is that telematics classes did not ensure their children’s real learning, so it did not make sense to carry out the assessment in the way it was usually carried out; instead, new formulas that are better adapted to the situation and real context should be sought out.

About the degree of satisfaction expressed by families regarding the management carried out by the educational centres, in general terms, it can be stated that the majority of families (71.3%) were satisfied with the management, considering the performance of the schools to be excellent 8.1% (*n* = 14), very good 25.3% (*n* = 44) or good 37.9% (*n* = 66). On the other hand, 19.5% (*n* = 34) said that the management was sufficient, while 8.6% (*n* = 15) said that the management was insufficient.

## 4. Conclusions

The pandemic generated by COVID-19 that spread through several countries during 2020, among many changes, gave an unexpected twist to the management of communication between schools and families. First, as set out in the *Guidance for schools and educational institutions in dealing with cases of COVID-19* [3], the task of reporting the disease and ways to stop its spread was added to the duties of teachers. Subsequently, teachers and school staff faced a new challenge, as the evolution of the pandemic made it necessary to close schools, and consequently, all communication between schools and families had to be conducted virtually.

In line with recent studies related to the management of education and COVID-19 [26], the family member who completed the questionnaire were mostly the mothers. This corroborated that, within the family environment, mothers continue to be the ones in charge of managing matters related to their children’s education. For nearly half of the families, it is again the mothers who are responsible for attending to and supervising their children’s homework. This issue is also shown in studies that research how women are normally those in charge of childcare at school-related matters, responsibility being an invisible task [27]. These results are in line with many studies on work–family conciliation where, once more, those who reduce their working hours to facilitate childcare responsibilities are women [28,29]. In the 21st century, where balancing work–family life is an open debate, co-responsibility between parents is still an issue that needs to be addressed in an equitable manner.

One interesting finding is that during the lockdown, families were obliged to pay more attention to their children’s homework since they were at home. In this way, the responsibility for the teaching–learning process, which normally lays on the teachers, was partially transferred to the family [7,8]. This has generated a series of difficulties that, although not new, have been accentuated by the situation. Thus, the greatest obstacle highlighted by families has been the lack of academic knowledge regarding the school subjects that their children have had to work on at home and feeling overwhelmed by the lack of specific knowledge of the subjects [22,25]. However, in many schools, teachers gave homework to students and resolved some questions by e-mail and other virtual channels [20]. Another important finding is that, even though communication channels between schools and families are essential [21], most of the time, since students are at home, parents were the ones dealing with these academic questions.

On the other hand, prior to lockdown, nearly half of the families stated that their children had received information on disease prevention measures based on the government guidelines [3,4]. However, almost a third of the families were not aware of whether their children had received instructions. Consequently, it is necessary to emphasize the importance of appropriate information management during a crisis that is based on credibility, trust, accountability and strategic organisation [13,15].

Data analysis regarding crisis communication management by the centres once their closing was required shows that, in a percentage of over 90%, families felt satisfied. In general, it can be stated that the families felt that they had been cared for and, although not all of them were clear about whether the schools had worked with the students on basic hygiene recommendations to prevent the spread of the disease, they agreed that they had been informed of other issues in a solvent manner, coinciding with the importance of communication between the family and the educational centre [20,21], as well as in relation to the quality of crisis communication [13,15]. In line with these findings, the communication conveyed by schools focused on two main topics: health and academic-related issues. This may seem logical, although, considering the situation of lockdown and the fact that most of the students were teenagers, it would have been necessary to include guidelines on mental health to avoid lockdown-linked negatives [11,30]. For this reason, in the event of a new crisis, it would be desirable to place mental health issues as a top priority. A possible starting point for this could be the framework to guide an educational response to the COVID-19 pandemic, stating that the school, among other functions, should provide support and strategies for good development of students’ mental health, especially during the lockdown [31].

Although the data referring to the general perception of families are very positive, a detailed study of their responses shows that, regarding communication management during the lockdown, no systematization was applied, and no guidelines were followed. Improving communication and reflecting on the instruments of information exchange between parents and teachers should be a priority [32] from a standpoint of relations characterized by “reciprocity” (equality of status) and “mutuality” (having issues in common).

For some schools, a wide variety of technological resources were employed to establish communication with families (email, educational platforms and instant messaging, among others), which have necessarily relied on the time and effort of teachers. The situation experienced during the health alert has generated teaching–learning models based fundamentally on technologies, where online teaching was fundamental for students and teachers [33]. In addition to this, information has been transmitted by different people in different positions within the educational teams (management, teachers, school psychologist and members of the AMPA). It is necessary to recognize the opportunity we have been given to develop other formulas for teaching–learning and communication between schools, always bearing in mind “[…] the training of teachers and students in digital competences, the transformation of teaching roles and guaranteeing social equity in access to technologies” [34].

The uniqueness of this pandemic and the sudden closing of schools led to the hasty implementation of measures that present room for improvement. In this respect, the present study can serve as a basis for the generation of guidelines and action models to provide better responses in the future. In this sense, there is a need for a technical figure trained to deal with this type of emergency, so a link between educational centres and families is created to act as a digital inclusion technician. A digital gap that not only involves access to and management of technological resources but also access to communication with the centres. We cannot forget that the main channels of communication used by the centres and families have been via e-mail or PASEN, and if families are not familiar with these resources, they may have been in a situation of unequal access opportunities. On the other hand, the autonomy of the centres to self-manage the resources is fundamental, allowing them to adjust to the needs of the environment, the families and the educational centre itself. This makes it possible to adapt the response in emergency situations to the different requirements of each educational community, thus optimising operability and successful results.

The major limitation of this study is that that the sample is not representative of the population of families with children attending Secondary Education in Andalusian schools. Notwithstanding this limitation, the data, results and conclusions of the present study establish the first step for more extensive studies using samples that represent the Andalusian population and even the Spanish one.

Among the aspects addressed to contextualise the pandemic situation presented in the introduction are several issues that have become evident. In the present study, they help us to understand the socio-educational situation in which the data have been collected. However, they allow us to lay the foundations for the extension of the study with future lines of research. Such lines of research could address: (1) mental health’s impact on adolescents in school; (2) *infodemics* in the socio-educational environment; (3) the impact of the digital divide on academic performance; and (4) the digital competences of teachers, students and families.

As a final conclusion, it can be affirmed that, despite the difficulties, the educational centres, according to the families’ perception, have offered a satisfactory response in terms of communication management. In this sense, the imbalance between face-to-face and virtuality generated by the schools closing revealed the inequality between favoured and disadvantaged environments based on the increased dependence on technological resources and the academic and digital literacy of families [22,25,35,36]. On this occasion, teachers and schools have used individual initiatives and efforts to address one of the most important educational challenges of recent decades. The schools made an extra effort to manage information in order to keep both families and students informed, and this was satisfactorily perceived by families. However, a percentage of around 10% of families considered that the crisis communication carried out by the educational centres was insufficient, which, considering the relevant role of families in accessing and monitoring their children’s education, is an aspect that should be improved in the future. In this sense, information and digital competences are key and essential for the everyday life of citizens, education, the workplace and even health. It would be advisable to establish guidelines to optimise efforts and cover all the necessary aspects in terms of information issues in emergency situations.

## Figures and Tables

**Table 1:** System of dimensions, codes, and item description.

Dimension	Code	Item Description
Informative or contextual	INFCO	Parent answering the survey (mother, father or guardian)
		Family circumstances during lockdown
		Number of children in secondary education
		Courses these children are inscribed in
		Type of educational centres
		Educational centres’ municipality
Crisis communication management	GCOMC	Preventive measures for COVID-19 infection conveyed by schools before lockdown
		Contact channel used by schools with their students’ families to inform them about the COVID-19 crisis
		Staff members who contacted the families
		Channels used to inform families
		Aspects that have been conveyed
		Families’ assessment of the communication management made by the centres
Education management	GESED	Prioritised school subjects for assignments
		Who is taking care of homework at home?
		Other activities taking place at home
		Families’ feelings about the activities developed at home
		Obstacles encountered by families in organising and implementing the educational routine proposed by the school
		Opinion of families on the amount of work requested by the school
		How children cope with time management when it comes to schoolwork
		Families’ perceptions of the learning-related information since lockdown
		Families’ views on their children’s assessment during the COVID-19 crisis

**Table 2:** Information received by families from the school.

Item	Frequency	Percentage
Yes, the school keeps us continuously informed.	80	45.9
Yes, the school has contacted us on an ad hoc basis.	75	43.1
No, it is the AMPA who has contacted us.	1	0.6
No, it is the parent delegate who is informing us.	1	0.6
No, the school has not informed us about this.	16	9.2
Do not know/No answer	1	0.6
Total	174	100

**Table 3:** Communication channels through which families have been contacted by the centres during the health crisis.

Item	Frequency	Percentage
Through the institutional educational application (PASEN)	113	30.7
Through an institutional e-mail	85	23.1
Through educational applications such as Google classroom, etc.	53	14.4
Via mobile instant messaging, WhatsApp, telegram, …	47	12.8
Through an email from the tutor or another teacher	29	7.9
Through the centre’s social networks, Twitter, Facebook, Instagram, etc.	24	6.5
Via personal email from a family member of the student	3	0.8
Through a phone call	2	0.5
Through a written statement hand-delivered to the student body	1	0.3
The family was not contacted by any means of communication	1	0.3
Do not know/No answer	10	2.7
Total	368	100

**Table 4:** Types of information provided by schools to families related to the health crisis.

Item	Frequency	Percentage
General information on the current health alert situation	95	20.4
Specific information on COVID-19 and its health implications	34	7.3
Basic recommendations on how to act at home (washing hands, etc.)	46	9.9
Information on academic homework to be done at home	135	29.0
Information on complementary educational platforms for training	77	16.5
Information on play activities your children can do at home	46	9.9
Information and strategies for maintaining good mental health	24	5.1
Administrative formalities and information on the continuation of the course	1	0.2
I have not received any information	1	0.2
Do not know/No answer	7	1.5
Total	466	100
